# Assessing *ZNF154* methylation in patient plasma as a multicancer marker in liquid biopsies from colon, liver, ovarian and pancreatic cancer patients

**DOI:** 10.1038/s41598-020-80345-7

**Published:** 2021-01-08

**Authors:** Brendan F. Miller, Hanna M. Petrykowska, Laura Elnitski

**Affiliations:** Translational and Functional Genomics Branch, National Human Genome Research Institute, National Institutes of Health, Bethesda, MD 20892 USA

**Keywords:** Diagnostic markers, DNA methylation

## Abstract

One epigenetic hallmark of many cancer types is differential DNA methylation occurring at multiple loci compared to normal tissue. Detection and assessment of the methylation state at a specific locus could be an effective cancer diagnostic. We assessed the effectiveness of hypermethylation at the CpG island of *ZNF154*, a previously reported multi-cancer specific signature for use in a blood-based cancer detection assay. To predict its effectiveness, we compared methylation levels of 3698 primary tumors encompassing 11 solid cancers, 724 controls, 2711 peripheral blood cell samples, and 350 noncancer disease tissues from publicly available methylation array datasets. We performed a single-molecule high-resolution DNA melt analysis on 71 plasma samples from cancer patients and 20 noncancer individuals to assess *ZNF154* methylation as a candidate diagnostic metric in liquid biopsy and compared results to *KRAS* mutation frequency in the case of pancreatic carcinoma. We documented *ZNF154* hypermethylation in early stage tumors, which did not increase in most noncancer disease or with respect to age or sex in peripheral blood cells, suggesting it is a promising target in liquid biopsy. *ZNF154* cfDNA methylation discriminated cases from healthy donor plasma samples in minimal plasma volumes and outperformed *KRAS* mutation frequency in pancreatic cancer.

## Introduction

Many cancer cases go undetected until patients develop symptoms, at which time the disease has often progressed to an advanced stage with poor treatment outcomes. For example, over half of patients with pancreatic cancer are diagnosed only after their disease has metastasized to sites distant from the original tumor, at which point the 5-year survival rate is only 3%^1^. A molecular screening test capable of detecting cancer could allow patients to be diagnosed even in the absence of signs and symptoms, which could lead to earlier treatment and thus improved survival rates. Liquid biopsies, which detect circulating tumor cells or DNA (ctDNA) within a patient’s blood, hold great promise as the basis for such molecular screening tests: they are minimally invasive, convenient, and could potentially detect many different types of cancer at once. In addition, liquid biopsies can be performed when tissue biopsies are not feasible—because of the position of a tumor within the body, for example—and they may better capture the scope of tumor heterogeneity than do tissue biopsies^2^. Before a liquid biopsy-based screening test is developed, however, suitable biomarkers must be developed. Here we test the suitability of one such candidate biomarker, DNA methylation at the *ZNF154* locus, for this purpose; we initially discovered methylation near the transcriptional start site of this this gene in a search for a multicancer marker^3,4^, and it has subsequently been implicated as a tumor suppressor^5^.

Historically, developing a ctDNA biomarker suitable for screening purposes has proven challenging. ctDNA-based liquid biopsies are already being used today as companion tests. For example, tests that detect *EGFR* mutations in ctDNA have been approved to guide therapy for non-small cell lung cancer^6^. Blood-based screening tests must pass a more rigorous hurdle, however, as they have to be able to detect the very low amounts of ctDNA present in blood samples at all stages of cancer, preferably including the very earliest stages, and be sufficiently sensitive and specific for the target disease^7^. Fortunately, increasingly sensitive technology is capable of detecting ever lower frequencies of ctDNA^8^, presenting new opportunities to increase the sensitivity of molecular screening tests if promising biomarkers are identified.

Recently, methylation signatures have been shown to correctly classify cancer versus normal tissue samples with high accuracy in many types of cancer^9,10^. Thus far in our analyses, *ZNF154* shows promise as a pan-cancer biomarker suitable for blood-based screening. We have shown that *ZNF154*, in particular, is methylated in 15 of 16 solid tumor types represented in the Cancer Genome Atlas (TCGA), whereas methylation levels in control samples are consistently low^4^. Moreover, our in silico analyses suggest that *ZNF154* methylation can be used to detect tumor signal in plasma samples as well, with a predicted AUC of up to 0.96 when ctDNA accounts for as little as 1% of cell-free DNA (cfDNA), for colon, lung, breast, stomach, and endometrial tumors^3^.

The next logical step is to see whether *ZNF154* methylation can be used to detect tumors of various types in actual patient plasma samples. In addition to verifying that *ZNF154* methylation is elevated in the plasma of patients with cancer, it is important to establish that methylation of this gene is not correlated with demographic factors such as age or sex, as previous studies have found for other loci^11–13^. It will also be important to confirm that *ZNF154* methylation is not elevated in the plasma of patients with non-cancer diseases. Thus, in this proof-of-concept study, we set out to determine whether *ZNF154* methylation is a suitable biomarker for a multi-cancer, plasma-based screening test. We show that *ZNF154* methylation is elevated in early-stage tissue samples from 10 different cancer types; is not meaningfully associated with age, sex, or non-cancer conditions; results in AUCs of up to 0.87 when used to identify plasma samples from cases versus healthy donors for multiple cancer types in the context of a liquid biopsy; and outperforms *KRAS* mutations as a plasma biomarker for pancreatic cancer. Notably, our *ZNF154* methylation detection method achieved 100% sensitivity and 80% specificity when used on plasma from patients with early-stage pancreatic adenocarcinoma, encouraging future studies to validate its effectiveness in early stage tumors.

## Methods

### Samples and datasets

#### *ZNF154* hypermethylation and mutation frequency in tumor samples

We analyzed *ZNF154* methylation at position cg21790626 in Illumina 450 K methylation array data derived from 3389 solid tumor samples from 10 solid tumor types and 27 K methylation array data from 302 ovarian carcinoma solid tumor samples, provided by The Cancer Genome Atlas (Table 1). We also analyzed mutation data for the same samples using information from cBioPortal^14^. Only TCGA tumor types with both methylation array data and mutation data available were further studied.

**Table 1:** Illumina 450 K and 27 K methylation array data for tumor versus control tissue samples.

Cancer type	Description	Controls	Tumors	Pathological stage I tumors
BLCA^b^	Bladder urothelial carcinoma	21	130	2
BRCA^b^	Breast invasive carcinoma	82	664	50
HNSC^b^	Head and neck squamous cell carcinoma	45	510	23
KIRC^b^	Kidney renal clear cell carcinoma	160	263	128
KIRP^b^	Kidney renal papillary cell carcinoma	45	267	70
LIHC^b^	Liver hepatocellular carcinoma	50	373	49
LUAD^b^	Lung adenocarcinoma	32	185	3
PAAD^b^	Pancreatic adenocarcinoma	10	150	21
PRAD^b^	Prostate adenocarcinoma	49	498	NA
STAD^b^	Stomach adenocarcinoma	2	349	2
OV^a^	Ovarian carcinoma	12	302	NA
Serous_EOC^c^	Serous epithelial ovarian carcinoma	216	7	7

^a^27 K methylation array data from TCGA.

^b^450 K methylation array data from TCGA.

^c^450 K methylation array data for serous subtype pathological stage I tumors (GSE72021)^15^ and fallopian tube controls (GSE74845)^16^.

#### *ZNF154* hypermethylation in early-stage tumor samples

We analyzed Illumina 450 K methylation array data from TCGA again, this time in stage I tumor samples from 9 different tumor types (BLCA, BRCA, HNSC, KIRC, KIRP, LIHC, LUAD, PAAD, STAD) and clinical stage I serous subtype epithelial ovarian carcinoma samples. In this analysis, we included pathological stage I tumors from the tumor types listed in Table 1, with the exception of prostate adenocarcinoma (PRAD) and ovarian carcinoma (OV) as there were no pathological stage I cases available. For early stage serous epithelial ovarian carcinoma, we included clinical stage I serous subtype ovarian carcinoma samples profiled by Illumina 450 K methylation arrays from Bartlett et al.^15^ (Serous epithelial ovarian carcinoma [Serous_EOC] Table 1, GEO accession GSE72021). Serous_EOC controls were fallopian tube tissue samples obtained from Bartlett et al.^16^ (GEO accession GSE74845).

#### *ZNF154* hypermethylation in tissue samples from individuals without cancer

We investigated associations between *ZNF154* methylation, age, and sex using TCGA Illumina 450 K human methylation array data from 2711 peripheral blood cell samples collected from patients without cancer (GEO accession GSE55763, described in^17^). We also analyzed *ZNF154* methylation in tissue samples collected from patients with disease conditions other than cancer, using Illumina 450 K human methylation array data from NCBI’s Gene Expression Omnibus (https://www.ncbi.nlm.nih.gov/geo/) and other sources (Table 2).

**Table 2:** Illumina 450 K methylation array data for tissue samples collected from donors without cancer.

Data source and/or publication	Description	Sample count
GSE42921^18^	Colon mucosa: normal controls	12
	Colon mucosa: Crohn’s disease	5
	Colon muscosa: ulcerative colitis	5
GSE81211^19^	Colon biopsy: normal	3
	Colon biopsy: ulcerative colitis	8
GSE85566^20^	Airway epithelial cells: asthma	74
	Airway epithelial cells: control	41
Dayeh et al.^21^	Pancreatic islets: type 2 diabetes	15
	Pancreatic islets: non-diabetic	34
GSE87621^22^	Endometriosis (cultured primary cells)	4
	Control (cultured primary endometrial stromal cells)	5
GSE49542^23^	Non-alcoholic fatty liver disease, mild (frozen liver biopsies)	35
	Non-alcoholic fatty liver disease, advanced (frozen liver biopsies)	24
GSE50874^24,25^	Kidney fibrosis	85

#### Counts of methylated *ZNF154* fragments in plasma samples from individuals with and without cancer assessed by DNA melt curve analysis

We analyzed *ZNF154* methylation in plasma samples purchased from Fox Chase Cancer Center, from 4 patients with colon cancer, 4 with liver cancer, 17 with pancreatic cancer, and 38 with ovarian carcinoma, as well as from 20 healthy donors without cancer. These samples encompassed approximately 1.5–4 mL (mLs) of plasma each and were obtained from donors with cancers at stage III and IV. An additional 8 plasma samples from patients with early-stage pancreatic cancer (1 stage I sample and 7 stage II samples) were also collected. Half of the available plasma for the pancreatic cancer samples and healthy donors was processed for use with DREAMing analysis^26^, whereas the remaining half was processed for *KRAS* mutation screening via ddPCR (see subsequent sections). Additional sample information can be found in Supplementary Table S1. All samples were obtained as commercial items and were covered under the Fox Chase Cancer Center Biorepository IRB review information.

### Data analyses

#### Illumina 450 K methylation array data analysis

Beta values were beta-mixture quantile (BMIQ) normalized^27^ and probes overlapping known SNPs were removed. Beta values were extracted for each sample from the previously identified *ZNF154* probe of interest (Illumina Infinium HumanMethylation450 (450 K) BeadChip array probe cg21790626).

#### Analysis of cancer gene mutation frequency

We obtained count data for cancer gene mutations from Genome Data Analysis Center (GDAC) Firehose Legacy files downloaded from cBioPortal (https://www.cbioportal.org/)^14^. For each cancer type, cancer-associated genes were defined as those that were mutated in > 10% of tumors based on cBioPortal “Mutated Genes” table and also present in the OncoKB database^28^. In the case of KIRP, no gene was mutated in > 10% of tumors so the top mutated gene was chosen instead. Thus, each cancer type assessed had its own set of cancer-associated genes. For a given cancer type, the proportion of tumors that contained a non-silent single nucleotide polymorphism mutation within one of the genes of the corresponding cancer type gene set was determined.

#### Analysis of cancer hypermethylation frequency

The frequency of tumors that were hypermethylated for a given cancer type was defined as the proportion of tumors whose beta value at cg21790626 was greater than a beta value cutoff at this site. The cutoff was defined as the beta value greater than 95% of the associated normal samples.

### cfDNA extraction

We performed cfDNA extraction and subsequent bisulfite conversion following previously published methods^29^ with the following modifications: the NeoGeneStar cfDNA Purification Kit with Pretreatment (NeoGeneStar, Somerset, NJ, USA) was used to extract cfDNA, which was bound to NeoGeneStar beads. Instead of eluting as per the standard protocol, we added 20 μL AE buffer to the 1.5 mL Eppendorf tube (Eppendorf, Hauppauge, NY, USA) containing the beads with bound cfDNA after the wash steps. From here, we then either proceeded with the Zymo Lightning Conversion kit (Zymo Research, Irvine, CA, USA) by adding 130 μL of conversion reagent to the 20 µL AE and bead solution (see below), or split the elution to perform ddPCR *KRAS* mutation screening on one half of the elution and bisulfite conversion in the remaining half via the Zymo Lightning Conversion Kit with spin columns following standard procedures.

### ddPCR in plasma samples

For the cfDNA elutions (see previous section) that were split, the elution half intended for ddPCR was first cleaned using Zymo DNA Clean and Concentrator-5 (Zymo Research, Irvine, CA, USA) using standard procedures and eluted twice in 10 μL of Zymo DNA Elution buffer. The elutions were then combined and DNA concentration was measured using the Invitrogen Qubit 3.0 Fluorometer and stored at − 80 °C.

To calculate the genomic copies of mutated *KRAS* present in cfDNA extracted from plasma samples, we used the BioRad ddPCR *KRAS* G12/G13 Screening Kit (which targets 7 different *KRAS* mutations G12A, G12C, G12D, G12R, G12S, G12V, G13D) (BioRad product number: 1863506) (BioRad, Hercules, CA, USA), using standard instructions with a C1000 Touch Thermo Cycler and the QX200 Droplet Generator and Reader System and an annealing temperature of 60 °C. 5 μL of a given sample elution was used as input for each reaction and at least two reactions for each sample were performed until the entire sample was used. Droplets were read on a QX200 Droplet Reader and data was analyzed using QuantaSoft software.

### Calculation of *KRAS* MtAF

The normalized *KRAS* mutant allele frequency for a given sample was calculated by first dividing the counts of mutant *KRAS* droplets detected by the total amplified droplets (wild-type plus mutant *KRAS*) measured for all ddPCR reactions for that sample. Then, these mutant allele fractions were adjusted by dividing by the estimated volume of plasma assayed in ddPCR for the sample. This was determined by multiplying the total starting volume of plasma for a given sample by the fraction of the elution that was used as input for the ddPCR assay. Sample plasma volumes assessed in ddPCR and MtAFs can be viewed in Supplementary Table S2.

### DREAMing analysis of *ZNF154* methylation

DREAMing is a highly sensitive DNA melt-based approach for detection of utra-rare methylated DNA fragments in patient samples such as blood plasma, as previously described^26,30,31^. In brief, this is achieved by assuming a bisulfite-converted sample containing a mix of epialleles (an epiallele being a sequence of DNA with a particular DNA methylation pattern), in which the methylated epialleles of interest are diluted in a background of the unmethylated epialleles. The sample is partitioned across many microtiter wells such that each well contains unmethylated background epialleles but the methylated epialleles will be distributed into only some of the wells based on a Poissonian distribution. PCR followed by high resolution melt analysis is performed and each well will exhibit one of two melt curves. Wells containing only unmethylated DNA will exhibit a single melt peak at a characteristic temperature, Tmu. On the other hand, wells that contain a methylated epiallele will exhibit 2 melt peaks: 1 from the unmethylated epialleles and 1 (higher-temperature) melt peak that is derived from the amplicons of the methylated epiallele. We can then determine the relative methylation density of each detected epiallele by observing the melt temperature of their respective secondary peaks, which will be proportional to the methylation density (number of methylated CpGs) of their respective template epiallele. As the frequency of the secondary melt peaks follow a Poissonian distribution, the methylation of each epiallele can be determined and quantitated at single copy sensitivity.

We bisulfite converted cfDNA from plasma samples by adding 130 μL Zymo Lightning Conversion Reagent to the 20 μL AE, cfDNA and NeoGeneStar bead solution in a 1.5 mL Eppendorf tube (see above, cfDNA extraction). For elutions that were split, volume was brought up to 20 μL with water before addition of the 130 μL Zymo Lightning Conversion Reagent. Samples were then incubated at 98 °C for 8 min followed by incubation at 56 °C for 1 h in the dark. 400 μL Zymo M-Binding was added to the samples, and the solution was gently mixed by pipetting, followed by incubation at room temperature for 5 min. Beads were gently spun down and tubes were placed onto a magnetic rack until the liquid cleared. Liquid was aspirated off, followed by addition of 400 μL Zymo Wash buffer, and subsequent steps followed the standard protocol for the Zymo Lightning Kit with Beads. Two elutions were performed, with beads incubated at 60 °C for 5 min with 50 μL Zymo Elution buffer each time, and each elution was stored separately at − 20 °C. cfDNA concentration measurements were preformed using TaqMan qPCR with primers specific to the 100 bp bisulfite-converted sequence of beta-actin and DREAMing reactions as previously described^26,30^.

Melt peaks of the products amplified in the DREAMing reactions were largely bimodal and represented either unmethylated or methylated DNA fragments with methylation densities equivalent to 1 or more methylated CpG sites. Counts of methylated melt peaks (i.e. proxies for detected fragments) were normalized by dividing by the equivalent volume of plasma loaded into the DREAMing assay for a given sample. The volume of plasma used for a sample assayed in DREAMing was determined from the cfDNA concentration by taking the fraction of bisulfite converted beta-actin targets loaded into all DREAMing wells for a given sample and multiplying the starting plasma volume by that amount. Please see Supplementary Table S1 for the equivalent volume of plasma and normalized counts of methylated *ZNF154* cfDNA per mL of plasma for each sample.

### DREAMing sensitivity

To test the sensitivity of the DREAMing assay targeting methylated cfDNA fragments of *ZNF154* we generated spike-ins of single molecules of fully methylated bisulfite converted mimetic synthetic *ZNF154* targets (Integrated DNA Technologies, Coralville, IA, USA) in a background of unmethylated bisulfite converted male genomic DNA (Promega, Madison, WI, USA). DREAMing primers: F: 5′-GGGCGATATTGGTAGGGATT-3′; R: 5′-AAATATATTCACCGAATCAAAAATAACAAAA-3′; 175 bp fully methylated mimetic: 5′-GGGCGATATTGGTAGGGATTCGGGGATAGCGGTTTTTATTTTAGGTTTGACGTGGGTTTTTTAGGGCGGCGTCGTTAAGGTTTAGACGTTTTCGTGTAGGAGGGACGACGATTTTTTTTACGTTTTCGTGGTTTTAATTCGGCGTTTTGTTATTTTTGATTCGGTGAATATATTT-3′. DREAMing reaction master mixes were designed as described above such that either 3 or 5 copies of fully methylated bisulfite converted mimetic synthetic *ZNF154* targets were expected to be diluted across 12 wells, with each well expected to have 200 copies of unmethylated bisulfite converted male genomic DNA. Each reaction was repeated a total of 5 times and the observed peaks of fully methylated bisulfite converted mimetic synthetic *ZNF154* were recorded. The mean absolute error for the measurement of detecting 3 or 5 copies of fully methylated bisulfite converted mimetic synthetic *ZNF154* target was calculated.

### Statistical analyses

Receiver operating characteristic curves, area under the curve (AUC) calculations, optimal cutoffs, and associated sensitivity and specificities were computed using python version 3.7.5 and the package sklearn 0.21.0.

## Results

### *ZNF154* hypermethylation and mutation frequency in tumor samples

On the basis of our previous results^3,4^, we hypothesized that *ZNF154* hypermethylation would occur more frequently in individual cancer types than the most common cancer mutations. To test this hypothesis, we compared the frequency of methylation at probe cg21790626 (the CpG site we had previously determined to be the best *ZNF154* methylation marker^3,4^) with the frequency of 52 mutations in cancer-associated genes for 11 solid tumor types from TCGA (N = 3691 for all tumor samples, N = 510 for all healthy tissue samples). Here, cancer-associated genes were defined as those present in the OncoKB database that were expected to be mutated in > 10% of samples for a given tumor type, based on cBioPortal mutation frequencies. For each tumor type, we established a *ZNF154* beta value hypermethylation cutoff that would exclude 95% of control samples. These beta value cutoffs values ranged from 0.016 to 0.523 with a median value of 0.161; using these cutoffs, we found that *ZNF154* was hypermethylated in 50.6–98.6% of tumor samples, depending on the tumor type (for example, 52% PRAD and 92.4% OV). For comparison, we used *TP53*, which was identified as a top recurrently mutated gene in all tumor types, except for KIRC and KIRP, in which only 3.0% and 1.9% of tumors contained *TP53* mutations. For the other 9 cancer types, *TP53* mutations—located at any position in the gene—were present in 9.8% (for PRAD) to 73.2% (for OV) of samples, depending on the tumor type. Thus, *ZNF154* hypermethylation at cg21790626 was present in approximately 1.5–26.6 times the number of tumor samples as *TP53* mutations, depending on the tumor type (Table 3).

**Table 3:** Frequency of tumors mutated or hypermethylated in cancer-associated driver gene sets for each cancer type.

Cancer type	Recurrently mutated genes selected in cancer-associated driver gene set for given cancer type^a^	Percent tumors mutated (%)^b^	Beta value hypermethylation cutoff^c^	Percent tumors hypermethylated (%)^d^
OV	*TP53* (73.2%)	73.2	0.313	92.4
BRCA	*PI3K3CA* (31.6%), *TP53* (24.8%), *CDH1* (7.1%)	54.2	0.190	74.4
PAAD	*KRAS* (90.7%), *TP53* (61.3%), *SMAD4* (16.7%), *CDKN2A* (10.7%)	95.3	0.070	90.7
LUAD	*TP53* (41.1%), *KRAS* (31.9%), *LRP1B* (25.4%), *PCLO* (18.4%), *STK11* (8.6%), *KEAP1* (15.1%), *RELN* (14.1%), *FAT4* (15.7%), *EGFR* (9.2%), *PTPRD* (13.5%), *CPS1* (10.3%), *GRIN2A* (10.8%), *NF1* (8.1%), *EPHA5* (9.2%), *FAT1* (10.8%), *MKI67* (8.6%), *SETBP1* (8.1%), *NOTCH4* (10.3%)	85.4	0.071	75.7
LIHC	*TP53* (24.1%), *CTNNB1* (25.2%), *ALB* (4.3%)	46.9	0.058	90.1
KIRP	*MET* (7.5%)	7.5	0.025	50.6
PRAD	*TP53* (9.8%), *SPOP* (11.0%)	20.5	0.523	52.0
HNSC	*TP53* (59.8%), *FAT1* (15.9%), *CDKN2A* (17.5%), *PIK3CA* (18.0%), *NOTCH1* (14.5%), *LRP1B* (17.1%), *KMT2D* (12.7%), *PCLO* (15.3%), *NSD1* (9.2%), *CASP8* (9.2%)	85.3	0.181	98.6
STAD	*TP53* (39.3%), *LRP1B* (24.6%), *ARID1A* (12.0%), *FAT4* (18.6%), *PCLO* (16.0%), *KMT2D* (10.0%), *PIK3CA* (16.9%), *ACVR2A* (1.4%), *LRRK2* (13.5%), *KMT2C* (8.6%), *CIC* (8.9%), *UBR5* (4.3%), *PREX2* (11.7%), *APC* (7.7%), *ERBB4* (11.5%), *TRRAP* (10.6%), *RNF213* (9.7%), *STK19* (0.3%), *KMT2B* (4.6%), *RPL22* (1.7%), *PTPRT* (8.9%), *PRKDC* (7.4%), *ZFHX3* (7.2%), *RELN* (9.7%), *EP400* (7.7%)	82.5	0.161	96.6
BLCA	*TP53* (45.4%), *ARID1A* (16.9%), *KDM6A* (15.4%), *PIK3CA* (20.0%)	63.8	0.316	94.6
KIRC	*VHL* (36.1%), *PBRM1* (19.8%), *SETD2* (9.5%)	52.9	0.016	75.3

^a^Percentages indicate the fraction of tumors from a given cancer type with a non-silent single nucleotide polymorphism in the given gene of the cancer-associated gene set.

^b^A tumor is considered mutated if it is mutated in any of the genes in the corresponding cancer-associated gene set.

^c^Beta value cutoff for a given cancer type at methylation array probe cg21790626 that is greater than 95% of the controls.

^d^Hypermethylation for each cancer type based on a beta value cutoff above 95% of the controls for that given cancer type.

**Figure 1:**
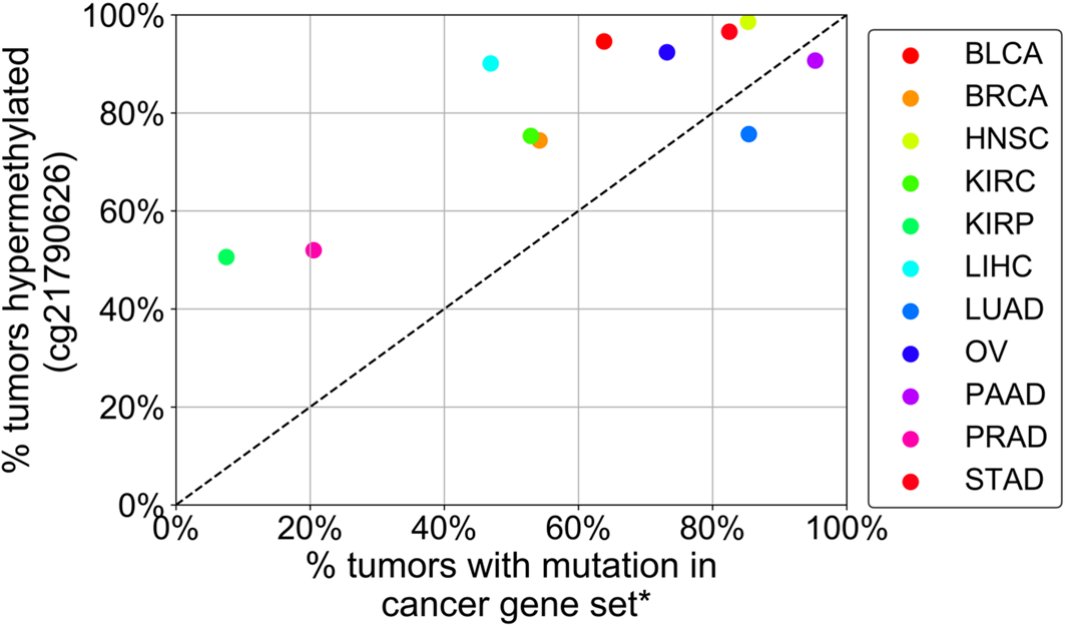
Percentage of tumors that displayed *ZNF154* hypermethylation at the probe cg21790626 versus percentage of tumors that displayed mutations in a set of cancer-associated genes for the given cancer type. The percent tumors with a mutation in a cancer gene set on the x-axis are the fraction of tumors that have a mutation in any of the cancer genes of the associated cancer gene set. Methylation data were obtained from The Cancer Genome Atlas and mutation data were obtained from cBioPortal. *A cancer-associated gene was included in a set if it was listed in the OncoKB database and expected to be recurrently mutated in > 10% of samples for a given tumor type, based on cBioPortal mutation frequencies. Lists of cancer associated genes for each cancer type are located in Table 3. *BLCA* bladder urothelial carcinoma (n = 130), *BRCA* breast invasive carcinoma (n = 664), *HNSC* head and neck squamous cell carcinoma (n = 510), *KIRC* kidney renal clear cell carcinoma (n = 263), *KIRP* kidney renal papillary cell carcinoma (n = 267), *LIHC* liver hepatocellular carcinoma (n = 373), *LUAD* lung adenocarcinoma (n = 185), *OV* ovarian carcinoma (n = 302), *PAAD* pancreatic adenocarcinoma (n = 150), *PRAD* prostate adenocarcinoma (n = 498), *STAD* stomach adenocarcinoma (n = 349).

Next, for each tumor type, we again utilized the *ZNF154* beta value hypermethylation cutoff sufficient to exclude 95% of control samples and compared the percentage of tumors with *ZNF154* hypermethylation with the percentage of tumors that harbored *any* cancer-associated gene mutation in the given cancer-type gene set (Fig. 1). The list of cancer-associated genes for each cancer type considered can be viewed in Table 3. *ZNF154* hypermethylation was less frequent than the combinations of cancer-associated gene mutations in only two cancer types. For example, in pancreatic adenocarcinoma samples we found 86.7% hypermethylated versus 95.3% mutated in the common set of PAAD cancer genes, (where 90.7% of tumors harbored a *KRAS* mutation and the remaining 4.6% had a mutation in another PAAD cancer gene: *TP53*, *SMAD4*, or *CDKN2A*). *ZNF154* hypermethylation was also less frequent than cancer-associated gene mutations in lung adenocarcinoma (75.7% hypermethylated versus 85.4% mutated in the common set of LUAD cancer genes), where most samples harbored either *KRAS* or *TP53* mutations (31.9% and 41.1% respectively). For all other tumor types investigated (9 out of 11), however, *ZNF154* hypermethylation was more common than cancer-associated gene mutations. The difference in recurrent hypermethylation versus mutations across tumors was particularly extreme in the case of kidney renal papillary carcinoma, in which 135 out of 267 tumors were hypermethylated but only 20 were mutated in a common set of cancer-associated genes. Thus, we concluded that *ZNF154* hypermethylation can be at least as recurrent as the most common cancer-associated mutations, making it a biomarker worthy of further investigation.

### Frequency of *ZNF154* hypermethylation in early-stage tumor samples

**Figure 2:**
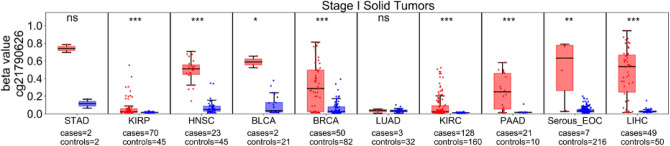
*ZNF154* methylation in stage I solid tumors. The probe cg21790626 was measured in 10 types of stage I solid tumor (red) or healthy tissue samples (blue), in Illumina 450 K methylation microarray data provided by TCGA or Bartlett et al. (Serous_EOC^15^, n = 7, and fallopian tube controls^16^, n = 216). Significant hypermethylation was found for all tumor types except for LUAD and STAD. “*ns*” not significant; *p < 0.05; **p < 0.01; ***p < 0.001; p values derived from Wilcoxon rank sum two-sided tests. Data plotted as standard box plot and whiskers. *BLCA* bladder urothelial carcinoma, *BRCA* breast invasive carcinoma, *HNSC* head and neck squamous cell carcinoma, *KIRC* kidney renal clear cell carcinoma, *KIRP* kidney renal papillary cell carcinoma, *LIHC* liver hepatocellular carcinoma, *LUAD* lung adenocarcinoma, *PAAD* pancreatic adenocarcinoma, *STAD* stomach adenocarcinoma, *Serous_EOC* serous subtype epithelial ovarian carcinoma.

The earlier that a biomarker can be detected in the course of tumorigenesis, the more helpful it will be for screening purposes. Thus, we next analyzed *ZNF154* methylation at probe cg21790626 in stage I samples, as available from 10 solid tumor types, as well as healthy control tissues, using Illumina 450 K methylation array data from either TCGA or serous epithelial ovarian carcinoma (Bartlett et al.^15^ [Serous_EOC] in Table 1, GEO accession GSE72021), and normal fallopian tube controls (Bartlett et al.^16^ GEO accession GSE74845). For all but 2 tumor types investigated, *ZNF154* was significantly hypermethylated with respect to control samples (*p* < 0.001; Fig. 2). The two exceptions were lung and stomach adenocarcinoma. For both of these tumor types, few cases were available for analysis (3 for the former, 2 for the latter); the data for stomach adenocarcinoma suggest that if more cases had been available for analysis as seen for bladder cancer, significant hypermethylation might have been detected at *ZNF154*.

### *ZNF154* methylation in tissue samples from individuals without cancer

**Figure 3:**
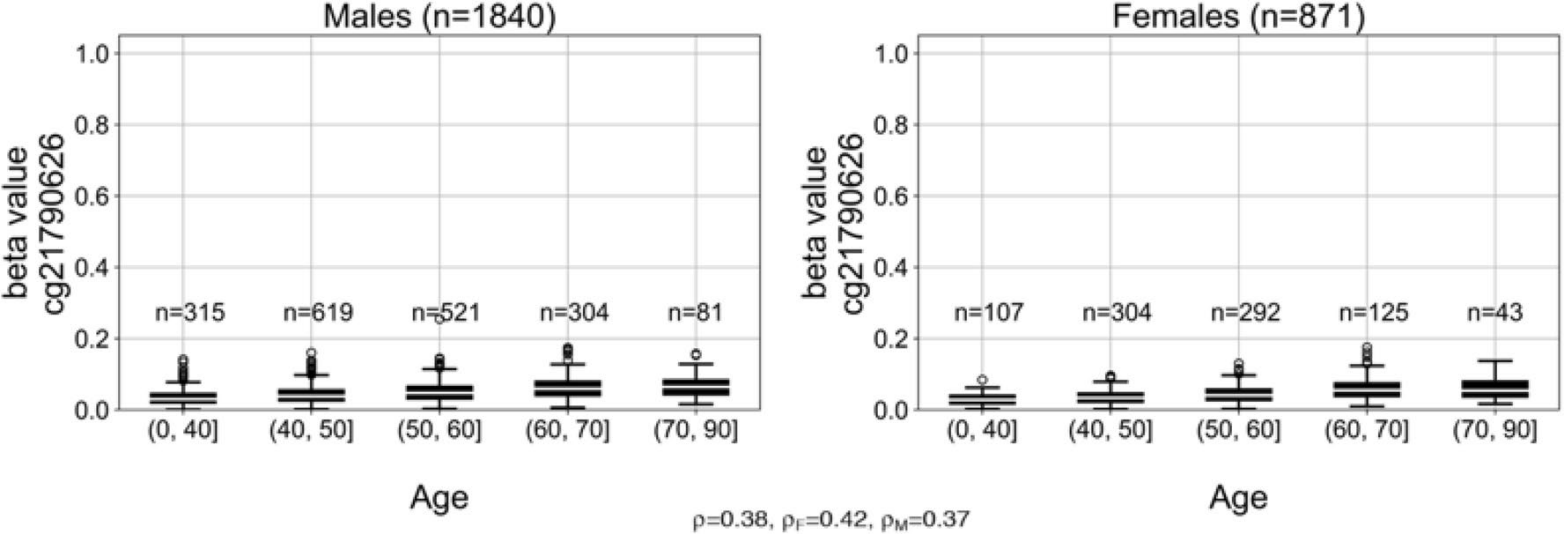
*ZNF154* methylation in 2711 peripheral blood cell samples collected from individuals without cancer, stratified by sex and age. Methylation was measured at probe cg21790626 in Illumina 450 K methylation array data provided by Lehne et al. (GEO accession GSE55763, described in^17^). Pearson correlation coefficients for beta value versus donor age for all samples (ρ), samples from only female donors (ρ_F_), or samples from only male donors (ρ_M_), are indicated below the figure panels. Data plotted as standard box plot and whiskers.

To be a viable screening biomarker, *ZNF154* methylation levels in cfDNA should not be associated with demographic factors such as age or sex. Most cfDNA in blood comes from circulating white blood cells^32^; thus, we tested the hypothesis that *ZNF154* methylation levels are not meaningfully elevated with age or sex by analyzing methylation at probe cg21790626 in TCGA Illumina 450 K methylation microarray data from 2711 circulating white blood cell samples (i.e., whole peripheral blood cell samples) from individuals without cancer. First, we stratified the samples by sex and partitioned them into bins based on the age of the peripheral blood sample donor. While we observed a positive correlation between the methylation level (beta value) at the probe of interest and patient age, no peripheral blood samples surpassed a beta value of 0.2 (female median beta value = 0.038; male median beta value = 0.042). Beta values below 0.2 are typically interpreted as a locus being lowly or unmethylated^33^, indicating that this genomic locus remains largely unmethylated even in older patients of either sex (Fig. 3). Therefore, assessing the methylation at *ZNF154* in a blood-based assay should not be expected to be compromised by noncancer conditions like patient age or sex.

**Figure 4:**
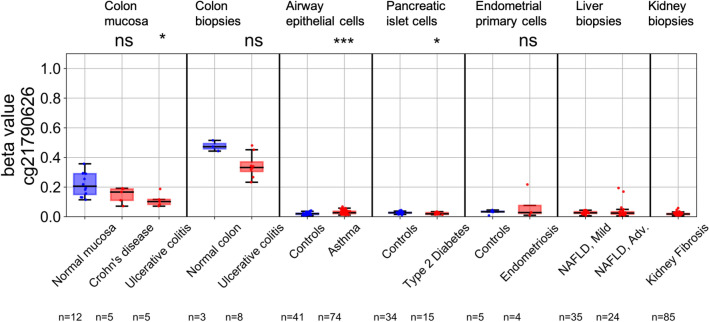
*ZNF154* methylation in 365 tissue and peripheral blood cell samples from individuals with and without various non-cancer conditions. Methylation was measured at probe cg21790626 in Illumina 450 K methylation array data from each dataset. *NAFLD* non-alcoholic fatty liver disease. Airway epithelial cells, pancreatic islet cells and ulcerative colitis showed significant differences between cases and controls (*p < 0.05, ***p < 0.001; *ns* not significant, two-sided Wilcoxon rank sum), with 332 samples showing beta values < 0.20.

To be a viable screening biomarker, *ZNF154* methylation levels should not be elevated in non-cancer health conditions; that is, methylation should be specific for cancer conditions and not elevated in other diseases, to avoid false positives. To test the hypothesis that *ZNF154* hypermethylation is limited to tumors, we analyzed *ZNF154* methylation at probe cg21790626 in array data from the following seven datasets (Table 2): (1) colon mucosa taken from patients with Crohn’s disease, ulcerative colitis, or healthy control individuals; (2) colon tissue taken from patients with ulcerative colitis or healthy controls; (3) airway epithelial cells in individuals with and without asthma; (4) pancreatic islet cells in individuals with and without type 2 diabetes; (5) endometrial primary cells in individuals with and without endometriosis; (6) liver biopsies in individuals with mild and advanced non-alcoholic fatty liver disease; and (7) kidney biopsies in individuals with kidney fibrosis (Fig. 4). For each noncancer set of disease samples with associated control samples, we assessed the difference in beta value distributions and observed significant differences only in ulcerative colitis with respect to colon mucosa (p < 0.05)—where disease samples showed lower methylation, asthma for airway epithelial cells (p < 0.001)—showing higher than normal methylation, and type 2 diabetes for pancreatic islet cells (p < 0.05)—also showing lower methylation than normals. Only in the case of asthma were the methylation levels at *ZNF154* significantly higher than that of the normal control epithelial cells. For reference, we compared the *ZNF154* methylation levels seen in control and tumor tissues from our preceeding TCGA analyses. Those beta value thresholds were required to exceed 95% of the TCGA normal controls to call tumors hypermethylated. There, a beta value threshold of 0.071 captured 95% of LUAD normal controls, and here the maximum beta value observed for the asthma samples was 0.068 (i.e., less than the TCGA normal control samples). Additionally, the methylation cutoff calculated for PAAD normal controls was 0.070 and the maximum beta value observed here for the type 2 diabetes samples was 0.034.

With respect to the normal colon mucosa and the colon tissue samples, the majority of beta values were above 0.2. This is consistent with our previous observation that the methylation level at *ZNF154* appears to be elevated in the gastrointestinal tract^4^. This suggests that DNA released from cells in the colon could contribute to a background methylation level present in the cfDNA pool at the *ZNF154* locus. Interestingly, with respect to the 450 K methylation array datasets shown here, we observed a decrease in methylation in samples from patients with either Crohn’s disease or ulcerative colitis in two independent studies (GSE42921 and GSE81211). Therefore, noncancer disease in the gastrointenstinal tract may actually decrease the methylation level at *ZNF154* and prevent detection of false positives. Nonetheless, only 18 of the 350 samples analyzed (5.14%) displayed a beta value ≥ 0.2. Thus, we concluded that, with rare exceptions, *ZNF154* is not hypermethylated (i.e., compared to normal) in non-cancer conditions we examined.

### Methylated *ZNF154* cfDNA levels in plasma samples from individuals with and without cancer

Having demonstrated that *ZNF154* methylation has the potential to be a promising multicancer marker in tissue samples, we wanted to investigate its use in plasma samples. We opted for an orthogonal approach toward detection of methylated cfDNA. This involved several components: (1) adaptation of a methylation on beads (MOB) extraction protocol for cfDNA^29^ to increase the efficiency of cfDNA recovery from plasma samples by a median of approximately 2.5-fold over other methods, and (2) adoption of a PCR-based high resolution DNA melt technique called DREAMing^26^ to identify rare, heterogeneously methylated DNA fragments in clinical samples. The method of DREAMing depends on sequence differences between methylated and unmethylated DNA molecules after bisulfite conversion and subsequent targeted amplification (where unmethylated “C”s become “T”s), which melt at different temperatures due to differences in base pairing energies. Multiple methylated CpGs in the amplicon additively increase the melt temperature peak. Primers to amplify our target *ZNF154* locus were designed for the DREAMing assay, such that they were preferentially biased toward amplification of methylated DNA (see “Methods”). The primers targeted a region that encompassed 14 internal CpG sites including the site assessed at cg21790626 by methylation arrays (Supplementary Figure S1). The sensitivity of the *ZNF154* DREAMing assay was tested by spike-in of single molecules of fully methylated bisulfite-converted mimetic synthetic *ZNF154* target into a background of bisulfite-converted gDNA from human male DNA (See “Methods”; Supplementary Figure S2).

We queried the methylation status of plasma derived *ZNF154* cfDNA fragments from a new cohort of patient plasma samples. These encompassed pancreatic (early-stage I, n = 1; early-stage II, n = 7; late-stage III–IV, n = 17), serous ovarian (late-stage III–IV, n = 38), liver (late-stage IV, n = 4), and colon cancer (late-stage IV, n = 4), as well as 20 healthy control donors (Supplementary Table S1). The median age of the control healthy donors was significantly higher than the ovarian and pancreatic cancer patient samples (controls median age = 71.5, ovarian cancer patient median age = 59.0, *p* < 0.001 Wilcoxon two-sided rank sum; pancreas cancer patient median age = 60.0, *p* < 0.01 Wilcoxon two-sided rank sum, Supplementary Figure S3). The older age of the controls may result in a higher background level of methylation at *ZNF154* in these samples. While this may effectively require using a more stringent threshold, this would also increase the confidence in any discriminating signal observed in the cancer patient cases as the methylation level would also need to be higher than any methylation potentially due to age.

**Figure 5:**
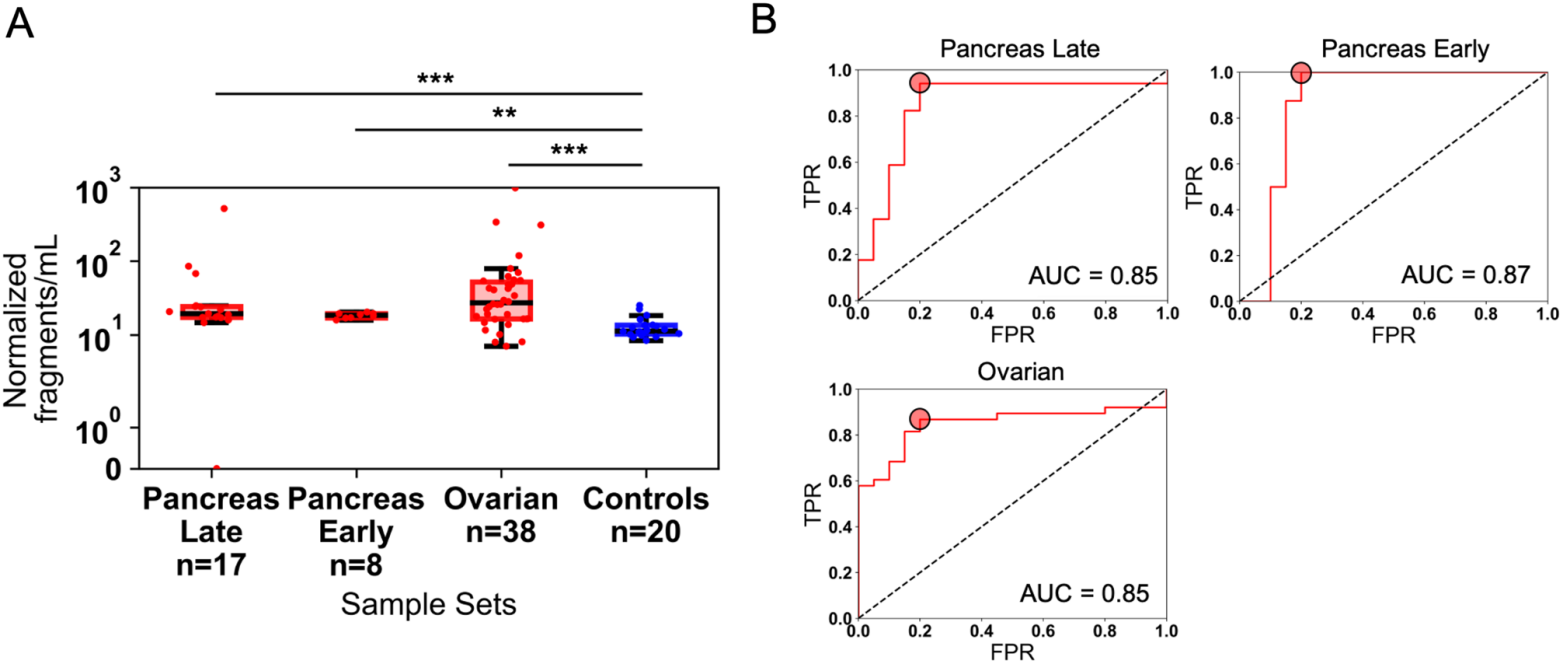
Measurement of methylated *ZNF154* fragments in plasma samples for cancer patient cases versus controls. A fragment is considered methylated if at least 1 of the 14 CpG sites measured per DREAMing amplicon is methylated. (**A**) Distribution of normalized methylated *ZNF154* cfDNA fragments per mL of plasma from patients with late-stage (stage III–IV, n = 17, red) pancreatic, early-stage (stage I–II, n = 8, red) pancreatic, or late-stage ovarian (stage III–IV, n = 38, red) cancer, or normal controls (n = 20, blue). Data are plotted using standard box and whisker plots. (**B**) Receiver operating characteristic curves showing the classification performance of the normalized fraction of methylated *ZNF154* cfDNA fragments per mL of plasma for the late-stage pancreatic, early-stage pancreatic, or ovarian cancer samples versus normal controls. Red circles indicate the optimal TPR and FPR combination based on the maximum positive difference between the TPR and FPR. ***p < 0.001; **p < 0.01, Wilcoxon rank sum two-sided test. *AUC* area under the curve, *FPR* false-positive rate, *TPR* true positive rate.

We took advantage of the single molecule sensitivity (quasi-quantitative nature) of the DREAMing assay, which allows for direct measurement of individual methylated fragment counts of cfDNA of interest, and tested the hypothesis that the concentration of methylated *ZNF154* fragments (i.e., fragments where we detected at least 1 methylated CpG site per DNA molecule) per mL of plasma could be helpful in classifying plasma samples from cases versus controls. Each sample set of cancer patient plasma had a significantly higher concentration of methylated *ZNF154* cfDNA fragments per mL than the normal controls (late-stage pancreatic cancer median normalized fragments = 19.47/mL plasma; ovarian cancer median normalized fragments = 27.30/mL plasma; normal controls median normalized fragments = 11.38/mL plasma) (Fig. 5A). Early-stage pancreatic cancer samples also had a significantly higher concentration of methylated *ZNF154* cfDNA fragments per mL of plasma than the normal controls (median normalized fragments = 18.6/mL plasma) suggesting that methylated *ZNF154* cfDNA may be an effective marker for detection of earlier stage cancers in liquid biopsies. With respect to the liver and colon cancer sample sets, each had higher median normalized fragments of methylated *ZNF154* cfDNA (14.56/mL and 64.49/mL, respectively) than the controls, although this was not significant, likely a result of the small size of these cohorts (Supplementary Figure S4).

We next derived optimal cutoffs based on the metric of normalized fragments of methylated *ZNF154* cfDNA per mL of plasma and used these to classify cases from controls for each sample set. We found optimal sensitivity/specificity ratios using receiver operating characteristic curve (ROC) plots, where we counted the total number of normalized methylated fragments for samples and controls. Optimal cutoffs were defined as those which maximized the positive difference between true positive rate and false positive rate (TPR–FPR). Overall classification performance of using the metric of normalized methylated fragments was measured as the area under the curve (AUC) of the ROC. We achieved AUC of 0.85 for late-stage pancreas (optimal sensitivity/specificity = 94.1%/80.0%), 0.87 for early-stage pancreas (optimal sensitivity/specificity = 100%/80%), and 0.85 for ovarian cancer (optimal sensitivity/specificity = 86.8%/80.0%) (Fig. 5B; Table 4). Although the sample size for the liver and colon cancer cohorts was small, we achieved optimal sensitivity/specificity ratios of 50.0%/90.0% and 75.0%/100.0%, respectively, suggesting *ZNF154* methylation may also be useful as a cancer marker in liquid biopsies from patients with these cancer types as well, although more extensive sample cohorts will be needed. Interestingly, the four normal controls with the highest normalized fragments per mL, and thus reducing the overall specificity based on the derived cutoffs, were also all above 80 years old, suggesting that age or conditions associated with age should be further studied for any effect on concentration of methylated *ZNF154* cfDNA in the circulation (Supplementary Table S1).

**Table 4:** Optimal cutoffs of normalized methylated *ZNF154* cfDNA fragments per mL plasma and the resulting classification AUCs, and optimal true positive rate (TPR) and false positive rate (FPR) for each plasma sample set.

Sample set	Cutoff (normalized fragments/mL plasma)	AUC	TPR	FPR	Sample size (tumor/normal)
Late stage pancreatic cancer	14.69	0.85	0.94	0.20	17/20
Early stage pancreatic cancer	15.96	0.87	1.00	0.20	8/20
Ovarian cancer	13.81	0.85	0.87	0.20	38/20
Liver cancer	21.89	0.48	0.50	0.10	4/20
Colon cancer	29.83	0.75	0.75	0.00	4/20

### Performance of *ZNF154* hypermethylation and KRAS mutation in classifying plasma samples from individuals with and without pancreatic cancer

Above, we found that *ZNF154* hypermethylation in Illumina methylation array data (90.7%) was as frequent as *KRAS* mutations (90.7%) for the PAAD samples (Table 3), making pancreatic cancer an interesting case study for comparing the utility of the two markers for classifying samples from blood. Finding an effective marker for detecting pancreatic cancer is also of special interest because this type of cancer is currently typically detected at a late stage, when outcomes are very poor. For these reasons, we decided to conduct a head-to-head comparison of *ZNF154* hypermethylation versus *KRAS* mutation for classifying plasma samples taken from individuals with and without pancreatic cancer.

**Figure 6:**
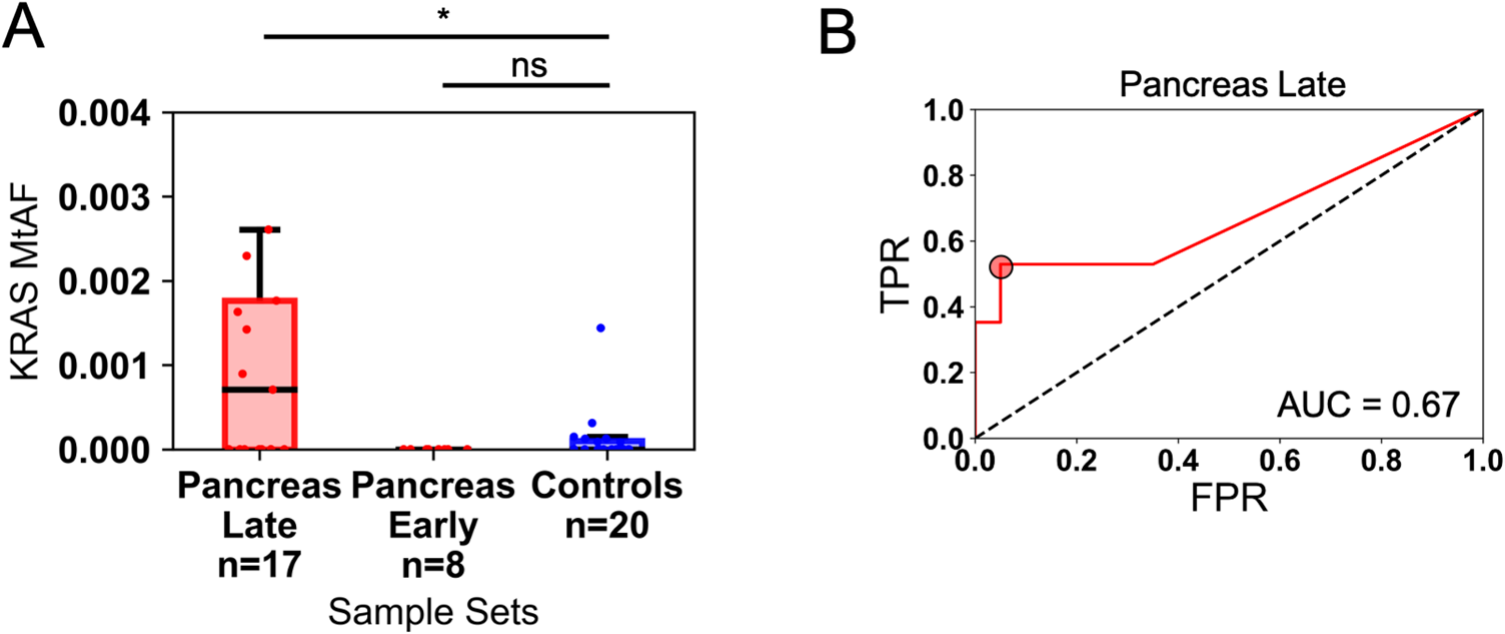
Classification performance of the *KRAS* mutant allele fraction (MtAF) in the cfDNA of 17 late-stage or 8 early-stage pancreatic cancer patient plasma sample cases and 20 healthy donors without cancer. (**A**) Distribution of *KRAS* MtAFs in cases and controls. Counts are plotted as a standard box and whiskers plot. Pancreas Late has 2 outliers not shown (see Supplementary Table S2 for full list of values). (**B**) Receiver operating characteristic curve for the classification of late-stage pancreatic cases and control donors using the sample *KRAS* MtAF. The red circle indicates the optimal MtAF cutoff based on the maximum positive difference between the TPR and FPR (optimal cutoff yielding 53% sensitivity/95% specificity). *p < 0.05; *ns* not significant, Wilcoxon rank sum one-sided test. *AUC* area under the curve, *FPR* false-positive rate, *TPR* true positive rate.

We experimentally analyzed *KRAS* mutations using ddPCR on the same 17 plasma samples from patients with late-stage pancreatic cancer, 8 patients with early-stage pancreatic cancer, and 20 individuals without cancer, using the same plasma input volumes used for the DREAMing analysis. We observed mutant *KRAS* fragments in 9 of 17 (52.9%) late-stage pancreatic cancer samples vs. 7 of 20 (35%) control samples after collectively targeting 7 different *KRAS* alterations in the cfDNA (Fig. 6; Supplementary Table S2). The late-stage pancreatic cancer cases had higher median *KRAS* mutant allele frequencies (MtAF) when normalized for mLs of plasma input (median MtAF = 7.07e−4; Supplementary Table S2 with respect to the controls (control median MtAF = 0.0; control maximum MtAF = 1.44e−3) and this difference was statistically significant (*p* = 0.036, Wilcoxon one-sided rank sum test). However, the sample set of 8 early-stage (I and II) pancreatic cancer plasma samples had no detectable *KRAS* mutant cfDNA. Using the *KRAS* MtAF cutoff to distinguish between late-stage case versus healthy donor plasma samples resulted in an AUC of 0.67 (optimal MtAF cutoff of 7.07e−4 yielded a sensitivity/specificity of 53%/95%). These findings, in comparison to our previous measurements of *ZNF154* cfDNA methylation (i.e., AUC = 0.85 at late stage), indicate that *ZNF154* has potential to outperform even the most common cancer-associated mutations when used to classify plasma samples from individuals with and without cancer. Moreover, *ZNF154* may be particularly helpful in detecting early-stage disease (AUC for early stage was 0.87), as shown for this small sample size of pancreatic cancers.

## Discussion

In this proof-of-concept study, we set out to determine whether *ZNF154* methylation appears to be a suitable biomarker for a multi-cancer, plasma-based screen**.** We found that *ZNF154* hypermethylation occurs in tissue samples from patients with early-stage tumors in 10 different cancer types; is not meaningfully associated with age or sex, or elevated in non-cancer conditions; and displays promising performance when used to classify plasma samples as tumor versus healthy donor controls, with AUCs of 0.85 for late-stage ovarian cancer, and 0.85 or 0.87 for late- or early-stage pancreatic cancer.

One key finding from our study was the importance of how methylation is quantified in plasma samples. We found that if *ZNF154* methylation was quantified using the average methylation levels in each sample, tumor signals in plasma often went undetected because of background signals in control samples. The *ZNF154* locus can be methylated in tissues of the gastrointestinal tract (see Figs. 2, 4), and it is possible that cfDNA derived from these tissues could contribute to this background methylation signal observed in healthy donor cfDNA. It will be important to establish a larger set of control samples to fully characterize the extent of background methylation and refine our classification cutoffs. In this study, the single-molecule sensitivity of the DREAMing DNA melting curve analysis used herein allowed us to use all methylated fragments of *ZNF154* cfDNA (heterogeneously methylated as well as fully methylated), which increased the amount of signal detectable and likely increased our ability to detect signal above background. For example, we found that we could only detect plasma samples for patients with early-stage pancreatic cancer reliably when we considered counts of all methylated cfDNA fragments. This finding contradicts the expected efficacy of a binary test like methylation-specific PCR.

The ability of *ZNF154* methylation to outperform *KRAS* mutations for the detection pancreatic cancer in patient plasma may reflect technical differences between the assays used and biological differences between DNA methylation and mutations. ddPCR and DREAMing are both are highly sensitive techniques and can detect rare DNA molecules robustly^26,31,34^. However, the targets of each assay are disparate and therefore difficult to directly compare. For ddPCR we utilized TaqMan probes to target 7 different *KRAS* point mutations. In contrast, for the DREAMing assay, we designed PCR primers to preferentially amplify methylated DNA of interest and subsequently performed a variation of DNA high resolution melt (HRM)^35^ to quantify the number of epialleles (i.e., DNA fragments from the same genomic location but with different methylation patterns) with different methylation densities (e.g., 1–14 internal CpGs methylated). It is important to note that this differs from other methylation detection assays like MSP, which only detect heavily or fully methylated epialleles^36^. By including *ZNF154* DNA fragments with any methylation detected, albeit heterogeneous or complete methylation, (i.e., any number of the 14 internal CpGs methylated), we were able to increase the number of methylated epialleles targetable for cancer detection and thus increased the ability to distinguish pancreatic cancer patients from healthy controls. Additionally, one of the shortfalls of the *KRAS* mutations was that these were present in 7/20 of the control samples, which lowered assay specificity. While methylated *ZNF154* fragments were also detected in the healthy controls, these levels were sufficiently low, enabling us to maintain high specificity, which is also consistent with the observation that *ZNF154* hypermethylation is an early event in cancers, including pancreatic cancer, and is also absent in noncancer conditions, as we show in this study.

*ZNF154’*s performance at identifying plasma samples from patients with cancer is competitive with that of other proposed biomarkers. For example, in this study, *ZNF154* methylation outperformed *KRAS* mutations in pancreatic cancer, when counts of any methylated *ZNF154* fragment were used to classify cancer versus control plasma samples. *ZNF154* methylation also should be compared with methylation of a recently proposed pan-cancer biomarker, Gasdermin E (*GSDME*)^37^. When *GSDME* was used to identify various tumor types based on methylation in tissue samples, its reported AUCs are 0.84–0.97; these values are similar to what we have reported previously for *ZNF154* in various tumor types (AUCS ≥ 0.95)^3^. However, *GSDME* methylation has not yet been tested in plasma samples, as *ZNF154* has in this study.

The preliminary results presented in this article suggest that *ZNF154* methylation also appears to be competitive with the multi-marker CancerSEEK test, a blood test that analyzes levels of 8 proteins and the presence of mutations at 1933 distinct genomic positions in cfDNA^38^. CancerSEEK’s reported median sensitivity across 8 cancer types was 70%, ranging from 98% in ovarian cancer to 33% in breast cancer. Its median sensitivity for stage I cancers was 43% overall, ranging from 20% for esophageal cancer to 100% for liver cancer. Finally, its overall specificity was ≥ 99%. Analysis of methylation at *ZNF154* is considerably simpler, cheaper, and faster than CancerSEEK, and achieves similar detection sensitivity with respect to colorectal and pancreatic cancer detection by only using a single genomic locus. Some of the proteins used by CancerSEEK are also elevated in people with inflammatory disease^39^, so the test’s false-positive rate is likely to be elevated in real-world populations, whereas *ZNF154* does not appear to be methylated in non-cancer conditions. While methylation array data derived from tissues of the gastrointestinal tract indicated elevated methylation at *ZNF154*, patients with Crohn’s disease or ulcerative colitis actually had reduced methylation levels relative to healthy controls. One important difference between *ZNF154* methylation and CancerSEEK is the ability of CancerSEEK to localize cancer to a small number of anatomic sites in a median of 83% of patients, which *ZNF154* methylation cannot do. Neverthless, given recent reports of elevated mutation rates stemming from clonal hematopeosis, the false positive rates of mutation screening will need to be carefully evaluated^40^.

In conclusion, our research indicates that *ZNF154* methylation testing in plasma may be a method capable of detecting multiple cancer types. This report provides proof of concept that *ZNF154* is a biomarker worthy of further study in the context of liquid biopsy-based lab testing or screening for cancer. However, although *ZNF154*′s performance in this study was satisfactory, it could also potentially be followed by additional markers that can help pinpoint the organ of origin for ctDNA, to aid diagnosis. Nonetheless, the ability of a single locus to achieve similar sensitivities to alternative methods that rely on hundreds or thousands of different markers suggests that the *ZNF154* assay presented here may be more applicable to the clinic. The assay itself is simpler, requires less patient material (we were able to detect discriminating signal with less than 2 mL of patient plasma), easier to implement (requires a qPCR machine and easily acquired reagents), and less expensive than current ultra-deep sequencing approaches.

In future studies, we plan to investigate *ZNF154* methylation in larger validation sets of plasma samples from patients with cancer of different types and stages, as well as larger control sample cohorts. In a previous study, *ZNF154* methylation was shown to be unhelpful for identifying thyroid tumors^4^, so it will be important to clarify which types of cancer the marker will miss. Eventually, it may be worthwhile to investigate *ZNF154* in a clinical trial, potentially as a marker used to screen patients at high risk of developing cancer^41^, providing that technical sensitivity can be achieved.

## Supplementary Information

Supplementary Information.

## Data Availability

Data from publicly accessible collections are listed in Table 2. The analysis code to reproduce the figures present here can be found at: https://github.com/bmill3r/ZNF154_Manuscript.

## References

[1] American Cancer Society (2019). Cancer Facts and Figures 2019.

[2] Tanić M, Beck S (2017). Epigenome-wide association studies for cancer biomarker discovery in circulating cell-free DNA: Technical advances and challenges. Curr. Opin. Genet. Dev..

[3] Margolin G (2016). Robust detection of DNA hypermethylation of ZNF154 as a pan-cancer locus with in silico modeling for blood-based diagnostic development. J. Mol. Diagn..

[4] Sanchez-Vega F (2013). Recurrent patterns of DNA methylation in the ZNF154, CASP8, and VHL promoters across a wide spectrum of human solid epithelial tumors and cancer cell lines. Epigenetics.

[5] Hu Y (2017). Candidate tumor suppressor ZNF154 suppresses invasion and metastasis in NPC by inhibiting the EMT via Wnt/beta-catenin signalling. Oncotarget.

[6] FDA approves first blood test to detect gene mutation associated with non-small cell lung cancer. https://www.fda.gov/news-events/press-announcements/fda-approves-first-blood-test-detect-gene-mutation-associated-non-small-cell-lung-cancer (2016).

[7] Alix-Panabières C, Pantel K (2016). Clinical applications of circulating tumor cells and circulating tumor DNA as liquid biopsy. Cancer Discov..

[8] Domínguez-Vigil IG, Moreno-Martínez AK, Wang JY, Roehrl MHA, Barrera-Saldaña HA (2017). The dawn of the liquid biopsy in the fight against cancer. Oncotarget.

[9] Hao X (2017). DNA methylation markers for diagnosis and prognosis of common cancers. Proc. Natl. Acad. Sci. USA.

[10] Zhang Y (2019). Screening dys-methylation genes and rules for cancer diagnosis by using the pan-cancer study. IEEE Access..

[11] Boks MP (2009). The relationship of DNA methylation with age, gender and genotype in twins and healthy controls. PLoS One.

[12] Day K (2013). Differential DNA methylation with age displays both common and dynamic features across human tissues that are influenced by CpG landscape. Genome Biol..

[13] Florath I, Butterbach K, Müller H, Bewerunge-Hudler M, Brenner H (2014). Cross-sectional and longitudinal changes in DNA methylation with age: An epigenome-wide analysis revealing over 60 novel age-associated CpG sites. Hum. Mol. Genet..

[14] Gao J (2013). Integrative analysis of complex cancer genomics and clinical profiles using the cBioPortal. Sci. Signal.

[15] Bartlett TE (2015). Intra-gene DNA methylation variability is a clinically independent prognostic marker in women's cancers. PLoS One.

[16] Bartlett TE (2016). Epigenetic reprogramming of fallopian tube fimbriae in BRCA mutation carriers defines early ovarian cancer evolution. Nat. Commun..

[17] Lehne B (2015). A coherent approach for analysis of the Illumina HumanMethylation450 BeadChip improves data quality and performance in epigenome-wide association studies. Genome Biol..

[18] Harris RA (2014). DNA methylation-associated colonic mucosal immune and defense responses in treatment-naive pediatric ulcerative colitis. Epigenetics.

[19] Kang K (2016). A genome-wide methylation approach identifies a new hypermethylated gene panel in ulcerative colitis. Int. J. Mol. Sci..

[20] Nicodemus-Johnson J (2016). DNA methylation in lung cells is associated with asthma endotypes and genetic risk. JCI Insight.

[21] Dayeh T (2014). Genome-wide DNA methylation analysis of human pancreatic islets from type 2 diabetic and non-diabetic donors identifies candidate genes that influence insulin secretion. PLoS Genet..

[22] Yotova I (2017). Epigenetic alterations affecting transcription factors and signaling pathways in stromal cells of endometriosis. PLoS One.

[23] Murphy SK (2013). Relationship between methylome and transcriptome in patients with nonalcoholic fatty liver disease. Gastroenterology.

[24] Ko YA (2013). Cytosine methylation changes in enhancer regions of core pro-fibrotic genes characterize kidney fibrosis development. Genome Biol..

[25] Bontha SV (2017). Effects of DNA methylation on progression to interstitial fibrosis and tubular atrophy in renal allograft biopsies: A multi-omics approach. Am. J. Transplant..

[26] Pisanic TR (2015). DREAMing: A simple and ultrasensitive method for assessing intratumor epigenetic heterogeneity directly from liquid biopsies. Nucleic Acids Res..

[27] Teschendorff AE (2013). A beta-mixture quantile normalization method for correcting probe design bias in Illumina Infinium 450 k DNA methylation data. Bioinformatics.

[28] Chakravarty D (2017). OncoKB: A precision oncology knowledge base. JCO Precis Oncol..

[29] Keeley B (2013). Extraction and processing of circulating DNA from large sample volumes using methylation on beads for the detection of rare epigenetic events. Clin. Chim. Acta.

[30] Miller BF (2020). Leveraging locus-specific epigenetic heterogeneity to improve the performance of blood-based DNA methylation biomarkers. Clin.. Epigenet..

[31] O'Keefe CM (2018). Facile profiling of molecular heterogeneity by microfluidic digital melt. Sci. Adv..

[32] Snyder MW, Kircher M, Hill AJ, Daza RM, Shendure J (2016). Cell-free DNA comprises an in vivo nucleosome footprint that informs its tissues-of-origin. Cell.

[33] Du P (2010). Comparison of Beta-value and M-value methods for quantifying methylation levels by microarray analysis. BMC Bioinform..

[34] Vessies DCL (2020). Performance of four platforms for KRAS mutation detection in plasma cell-free DNA: ddPCR, Idylla, COBAS z480 and BEAMing. Sci. Rep..

[35] Wojdacz TK, Dobrovic A, Hansen LL (2008). Methylation-sensitive high-resolution melting. Nat. Protoc..

[36] Herman JG, Graff JR, Myohanen S, Nelkin BD, Baylin SB (1996). Methylation-specific PCR: A novel PCR assay for methylation status of CpG islands. Proc. Natl. Acad. Sci. USA.

[37] Ibrahim J, Op de Beeck K, Fransen E, Peeters M, Van Camp G (2019). The gasdermin E gene has potential as a pan-cancer biomarker, while discriminating between different tumor types. Cancers (Basel).

[38] Cohen JD (2018). Detection and localization of surgically resectable cancers with a multi-analyte blood test. Science.

[39] Kaiser J (2018). 'Liquid biopsy' for cancer promises early detection. Science.

[40] Razavi P (2019). High-intensity sequencing reveals the sources of plasma circulating cell-free DNA variants. Nat. Med..

[41] Bardelli A, Pantel K (2017). Liquid biopsies, what we do not know (yet). Cancer Cell.

