# Supplementary material for: Assessing ZNF154 methylation in patient plasma as a multicancer marker in liquid biopsies from colon, liver, ovarian and pancreatic cancer patients
Source: Sci Rep. 2021 Jan 8;11:221. doi: 10.1038/s41598-020-80345-7 (PMC7794477; doi:10.1038/s41598-020-80345-7)
Supplement: Supplementary file 1 — Supplementary Information. [file 41598_2020_80345_MOESM1_ESM.docx]

**Assessing *ZNF154* methylation in patient plasma as a multicancer marker in liquid biopsies from colon, liver, ovarian and pancreatic cancer patients**

**Brendan F. Miller^1^, Hanna M. Petrykowska^1^, Laura Elnitski^#^**

^1^National Human Genome Research Institute, National Institutes of Health, Bethesda MD 20892

^#^Corresponding Author

**Correspondence to:** Dr. Laura Elnitski, Translational and Functional Genomics Branch, National Human Genome Research Institute, National Institutes of Health, Bethesda MD 20892. E-mail: [elnitski@mail.nih.gov](mailto:elnitski@mail.nih.gov)

CCGGGAGCGACGGGCGACATTGGTAGGGACCCGGGGACAGCGGTCCCTATCCCAGGCCTG

:++||||++|++||++|:|||||||||||::++||||:||++||:::|||:::|||::||

TCGGGAGCGACGGGCGATATTGGTAGGGATTCGGGGATAGCGGTTTTTATTTTAGGTTTG

ACGTGGGTCCCCCAGGGCGGCGTCGCCAAGGCTTAGACGCTTTCGTGCAGGAGGGACGAC

|++|||||:::::||||++|++|++::||||:|||||++:|||++||:||||||||++|+

ACGTGGGTTTTTTAGGGCGGCGTCGTTAAGGTTTAGACGTTTTCGTGTAGGAGGGACGAC

GACTCCCCTCACGCCTTCGTGGCCCCAACTCGGCGCTCTGCTATCTCTGATCCGGTGAAC

+|:|::::|:|++::||++|||::::||:|++|++:|:||:|||:|:||||:++|||||:

GATTTTTTTTACGTTTTCGTGGTTTTAATTCGGCGTTTTGTTATTTTTGATTCGGTGAAT

ACACCTCAGAGAAGCTAAAATGGCCGCCACGAAGAGGCCCCCCCAAAAGTCCCGTCCTTT

|:|::|:|||||||:||||||||:++::|++||||||:::::::||||||::++|::|||

ATATTTTAGAGAAGTTAAAATGGTCGTTACGAAGAGGTTTTTTTAAAAGTTTCGTTTTTT

**Supplementary Figure S1.** Sequence of *ZNF154* genomic region targeted in DREAMing. The “top strand” is the sequence of the top strand of the non-bisulfite converted genome. The “bottom strand” is the top strand of the bisulfite converted genome. Yellow highlighted sequences indicates the annealing positions of the DREAMing *ZNF154* primers. Gray or red highlighted dinucleotide sequences indicate the locations of the 14 internal CpG positions assessed in DREAMing. The red highlighted sequence is the CpG site assessed by Illumina 450K methylation array probe cg21790626. “++” = CpG dinucleotides protected from bisulfite conversion; “:” = presumed unmethylated C’s converted during bisulfite treatment. “|” = unchanged nucleotide bases.

**
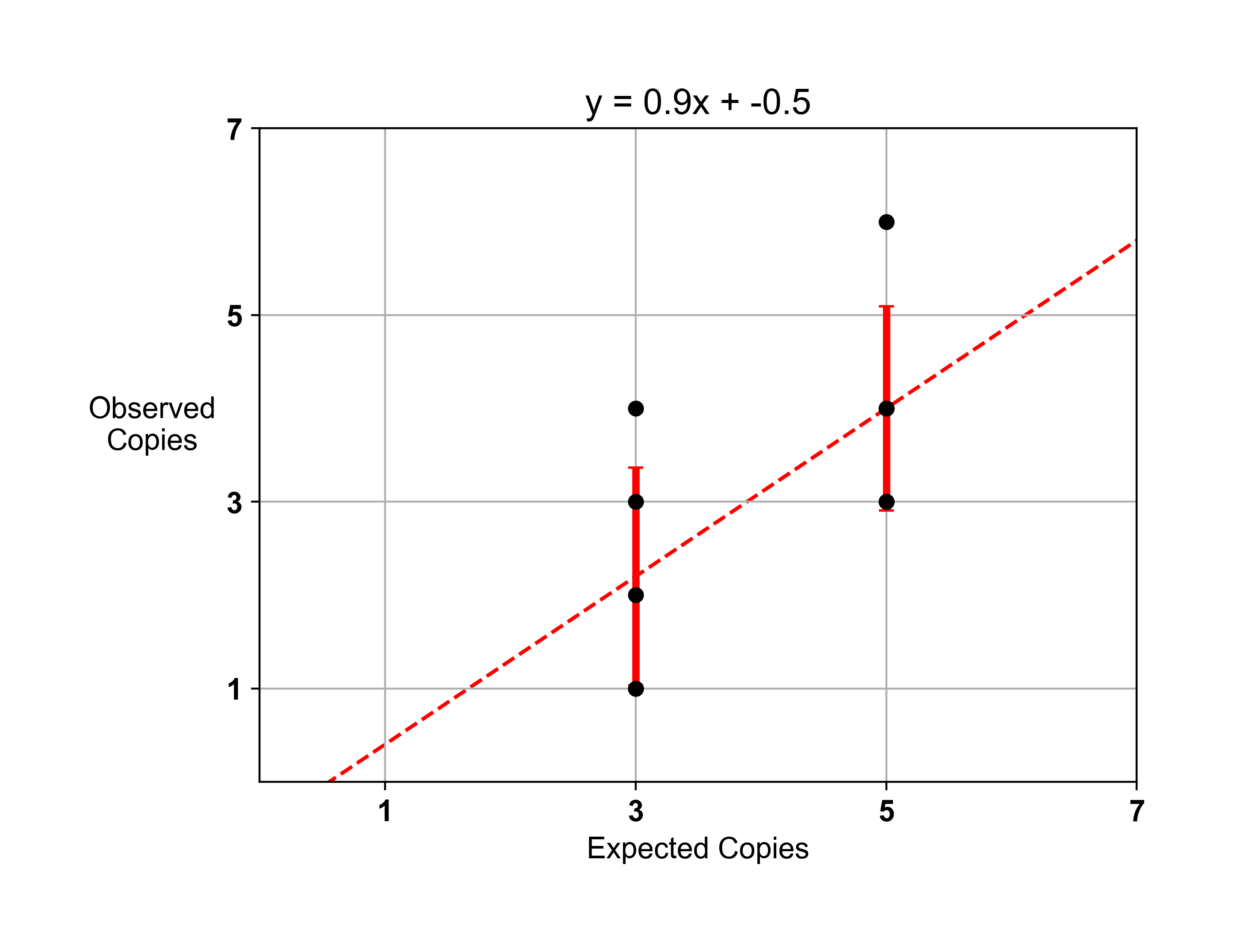
**

**Supplementary Figure S2**. Single copy sensitivity of DREAMing assay against fully methylated *ZNF154* fragments of DNA. The equation of the linear model is indicated at the top of the panel. Error bars indicate mean absolute error.

**Supplementary Figure S3**. Age distributions of patient cohorts. “ns” = not significant; ** = p < 0.01; *** = *p* < 0.001; p-values derived from Wilcoxon rank sum 2-sided tests. Data plotted as standard box plot and whiskers. Controls median age = 71.5; Ovarian median age = 59.0; Pancreas median age = 60.0; Liver median age = 61.5; Colon median age = 64.0.

**Supplementary Table S1.** Plasma samples used in study and associated patient information.

For each sample, the mLs of plasma extracted, copies of beta actin target after bisulfite conversion of the cfDNA, copies of beta actin loaded in to the DREAMing assay, and the relative volume of plasma assessed are listed. Sample barcodes are from Fox Chase Cancer Center.


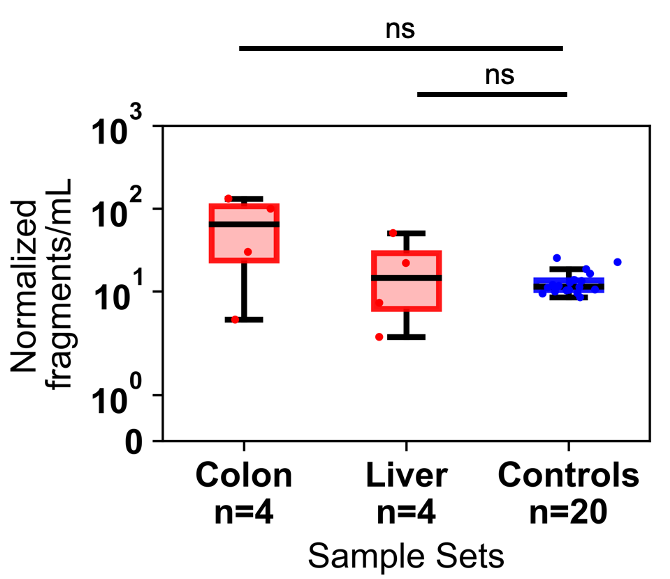


**Supplementary Figure S4**. Distribution of normalized methylated *ZNF154* cfDNA fragments per mL of plasma from patients with colon (n=4, *red*) or liver (n=4, *red*) cancer or normal controls (n=20, *blue*). Data are plotted using standard box and whisker plots. ns = not significant, Wilcoxon rank sum 2-sided test.

Supplementary Table S2. Pancreatic cancer and control donor adjusted *KRAS* MtAF.

MtAF = KRAS mutant copies / (KRAS WT copies = KRAS mutant copies); Adjusted MtAF/mL plasma = MtAF/mLs plasma assayed.
